# Quantifying the impact of small molecule ligands on G-quadruplex stability against Bloom helicase

**DOI:** 10.1093/nar/gkz803

**Published:** 2019-09-23

**Authors:** Parastoo Maleki, Golam Mustafa, Prabesh Gyawali, Jagat B Budhathoki, Yue Ma, Kazuo Nagasawa, Hamza Balci

**Affiliations:** 1 Department of Physics, Kent State University, Kent, OH 44242, USA; 2 Department of Biotechnology and Life Science, Tokyo University of Agriculture and Technology, Koganei, Tokyo 184-8588, Japan

## Abstract

G-quadruplex (GQ) stabilizing small molecule (SM) ligands have been used to stabilize human telomeric GQ (hGQ) to inhibit telomerase activity, or non-telomeric GQs to manipulate gene expression at transcription or translation level. GQs are known to inhibit DNA replication unless destabilized by helicases, such as Bloom helicase (BLM). Even though the impact of SM ligands on thermal stability of GQs is commonly used to characterize their efficacy, how these ligands influence helicase-mediated GQ unfolding is not well understood. Three prominent SM ligands (an oxazole telomestatin derivative, pyridostatin, and PhenDC_3_), which thermally stabilize hGQ at different levels, were utilized in this study. How these ligands influence BLM-mediated hGQ unfolding was investigated using two independent single-molecule approaches. While the frequency of dynamic hGQ unfolding events was used as the metric in the first approach, the second approach was based on quantifying the cumulative unfolding activity as a function of time. All three SM ligands inhibited BLM activity at similar levels, 2–3 fold, in both approaches. Our observations suggest that the impact of SM ligands on GQ thermal stability is not an ideal predictor for their inhibition of helicase-mediated unfolding, which is physiologically more relevant.

## INTRODUCTION

G-quadruplex (GQ) stabilizing small molecule (SM) ligands affect cellular biology in different ways. SM ligands that stabilize telomeric GQ structures (GQs) inhibit telomerase activity (1,2) and have the potential to diminish cancer proliferation (3). On the other hand, stabilizing GQs in regulatory sites, such as promoters in DNA or untranslated regions in RNA, enables gene expression regulation at transcription (4–6) or translation level (7). Large-scale screening studies have identified promising SM ligands that have high specificity for GQs and result in a large increase in GQ thermal stability, as measured by the change in thermal melting temperature (Δ*T*_m_) (8–11). Pyridostatin (PDS), PhenDC_3_, telomestatin (12,13), and oxazole telomestatin derivatives (OTD) are among the most promising such SM ligands (14–17).

SM ligands stabilize GQs to different degrees and Δ*T*_m_ has been typically used to quantify their efficacy. To that end, thermal melting assays based on Förster resonance energy transfer (FRET), circular dichroism (CD) or ultraviolet (UV) absorption are commonly used. To illustrate, Δ*T*_m_ ≈15°C was measured for human telomeric GQ (hGQ) due to 6OTD or 7OTD variants using both FRET-melting and CD melting assays at 100 mM K^+^ (18,19). On the other hand, Δ*T*_m_ values for PDS and PhenDC_3_ show a greater variation among different assays. At saturating ligand concentration, PhenDC_3_ resulted in Δ*T*_m_ = 29.7°C (at 100 mM Na^+^) (20), and PDS in Δ*T*_m_ = 35°C (at 60 mM K^+^) (21) for hGQ using FRET-melting assay. On the other hand, CD-melting resulted in Δ*T*_m_ = 8.8°C and Δ*T*_m_ = 14.4°C for PDS and PhenDC3, respectively; while UV-melting resulted in Δ*T*_m_ = 5.5°C and Δ*T*_m_ = 5.3°C for PDS and PhenDC3, respectively (22). In this same study, the authors performed FRET-melting measurements as well but concluded that the fluorescence quenching by PDS and PhenDC_3_ prevents reliable determination of *T*_m_ (22). These latter measurements were performed at 10 mM K^+^ and pH 5.7, which is lower than the pH 7.0–7.5 range typically used in FRET-melting assays. However, *T*_m_ for hGQ was demonstrated to be independent of pH in the pH 4.5–7.5 range (23). Another CD melting study showed Δ*T*_m_ = 6°C for PDS (10 mM K^+^ and pH 7.6) (24), in agreement with these lower Δ*T*_m_ values. We also performed CD-melting studies on hGQ with and without each of the three SM ligands and obtained lower Δ*T*_m_ values, in agreement with the latter studies (Supplementary Figure S1). In addition, these SM ligands are all highly selective to GQs compared to double stranded (dsDNA) or single stranded DNA (ssDNA). As the temperature is stable in cellular context, GQ destabilization is often facilitated by proteins, including ssDNA binding proteins and helicases (25,26). Therefore, how SM ligands affect the stability of GQs against these proteins is of fundamental significance; however, whether ΔT_m_ would be a reliable predictor for that is unclear.

GQ-specific SM ligands are typically used to establish the relevance of GQs in the cellular setting as their inclusion results in more stable GQs that might alter gene expression levels (27) or inhibit proteins from resolving them, which typically results in elevated levels of DNA damage and reduced proliferation (28). Some of the earliest *in vitro* work studied the inhibition of intermolecular GQ unfolding activities of human RecQ helicases Bloom (BLM) and Werner (WRN) by acridine derivatives (29) or porphyrin derivatives (30). These bulk biophysical studies demonstrated significant inhibition of helicases by these ligands, with ligand inhibition constants in the micromolar range. While the inhibition was reported to be GQ specific for the porphyrin derivative *N*-methylmesoporphyrin IX (NMM) (30), similar levels of helicase inhibition were observed for dsDNA and GQ for trisubstituted acridine derivatives (29). More recently, several studies have quantified the impact of SM ligands on helicase-mediated intramolecular GQ unfolding. Mendoza *et al.* utilized an ensemble fluorescence quenching assay to quantify the impact of several ligands (PDS, PhenDC_3_, Braco 19 and TrisQ) on Pif1-mediated unfolding of different intramolecular GQs (31). With the exception of TrisQ, which demonstrated very little to no inhibition of Pif1, the other ligands reduced Pif1 activity by up to several folds. Interestingly, hGQ was the most resilient among the nine constructs studied in this work. For hGQ, Pif1 activity in the presence of SM ligands was 40–60% of its activity in their absence. This resilience was attributed to polymorphism in the folding conformations of hGQ. Single-molecule investigations have also made significant contributions to our understanding of helicase, GQ and SM investigations. Based on SM-induced quenching of fluorescence, Tippana *et al.* suggested that RHAU helicase (also known as DHX36 and G4R1) easily displaces Braco19, NMM, and PhenDC_3_ before it repetitively unfolds the GQ (32). In this scenario, it was not clear how SM ligands inhibit helicase activity as the helicase easily displaced the ligands. The authors suggested using lower than typical ligand concentration in their assay, 50–100 nM instead of 1–5 μM, as a possible explanation.

Deciphering the underlying mechanism of helicase-mediated GQ unfolding would be an important step towards attaining a comprehensive understanding how SM ligands inhibit helicase activity. Even though SM ligands were not incorporated, two recent studies reported the structure of bacterial RecQ helicase bound to a folded GQ (33) and bovine RHAU helicase bound to a resolved GQ (34). The first study demonstrated that a guanine that was part of the GQ was flipped out and sequestered in a guanine specific pocket in RecQ, which was proposed as a potential mechanism for helicase-mediated GQ unfolding. In the second study, the authors proposed that binding of RHAU to GQ face and backbone induces ATP independent rearrangements in the helicase core. These rearrangements and rotation of C-terminal domain were proposed to be transduced into a directed pull on the ssDNA overhang neighboring GQ, resulting in remodeling of GQ and its unfolding one residue at a time. Whether these proposed mechanisms are applicable to other GQ resolving helicases and whether they could be reconciled into a more general mechanism remain open questions.

In this study, we utilized BLM as a model system to investigate the impact of SM ligands on helicase-mediated hGQ unfolding. BLM is a member of the RecQ family, which is involved in various aspects of genome maintenance (35,36). Bloom syndrome, due to deficiencies in BLM, is marked by genomic instability and increased predisposition to various cancers (37–39). Even though BLM can form a hexameric ring (40), BLM mutants lacking oligomerization domain have been shown to unfold dsDNA (41,42) and GQs (43,44) in 3′-to-5′ direction with low processivity. It has been suggested that the multimeric structures formed by BLM dissociate upon ATP hydrolysis and BLM monomer is responsible for unwinding dsDNA and unfolding non-canonical structures such as GQ (45). Cells deficient in BLM or other helicases with GQ unfolding activity, such as Pif1, demonstrate elevated levels of DNA breaks in potentially GQ forming segments of the genome and severe retardation of replication (46–48), highlighting the significance of effective removal of GQs by these helicases. Therefore, it is critical to understand the level of stability imparted by these SM ligands on GQs against helicases that are tasked to unfold them.

## MATERIALS AND METHODS

### Protein and DNA constructs

BLM^642–1290^ that maintains both dsDNA unwinding and ssDNA translocation activities was utilized in this study. This construct consists of RecQ core of BLM containing the helicase, RecQ C-terminal (RQC) domain and helicase and RNase D C-terminal (HRDC) domain (41).

**Figure 1.**
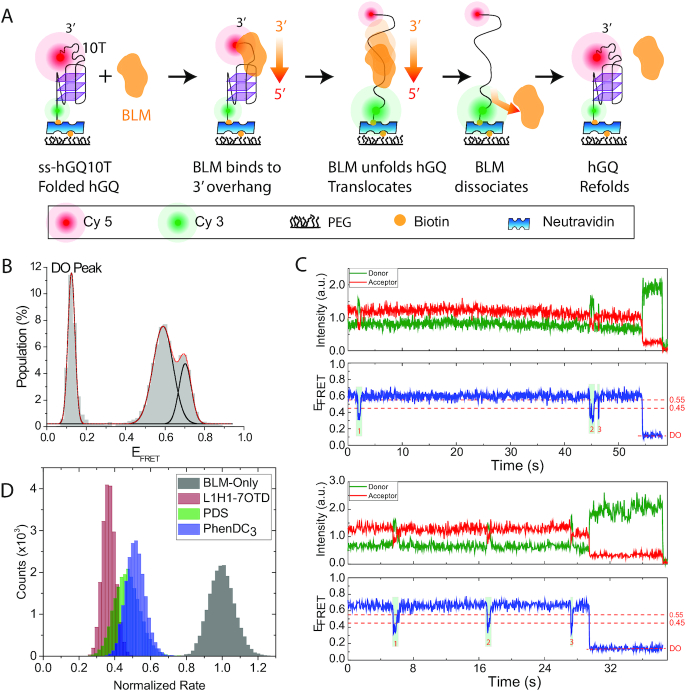
Quantifying the impact of SM ligands on BLM-mediated hGQ unfolding based on analysis of dynamic interactions. (**A**) A schematic of different steps of the BLM-mediated hGQ unfolding are illustrated. Donor (green) and acceptor (red) fluorophores are placed across hGQ. BLM binds to the 3′-overhang, translocates towards 5′-end, and unfolds the hGQ before dissociating from the DNA, allowing hGQ to refold. (**B**) The smFRET histogram showing folded state peaks at *E*_FRET_ = 0.70±0.03 and 0.59±0.05 at 150 mM KCl and 2 mM MgCl_2_. The peak at *E*_FRET_ = 0.12±0.02 represents the DO state, resulting from hGQ molecules without Cy5. The solid black curves are individual Gaussian fit peaks and the red curve is cumulative of three Gaussian peaks. (**C**) Two smFRET traces showing unfolding events starting from different folded FRET levels. The unfolding events are marked with green boxes and numbered. *E*_FRET_ = 0.45 and 0.55 (red dashed lines) represent the threshold FRET levels required for hGQ unfolding and refolding, respectively. An unfolding and refolding event has a signature that a high FRET state is followed by a dip to *E*_FRET_≤0.45 and a rise to *E*_FRET_≥0.55. (**D**) BLM-mediated hGQ unfolding rate without SM and with 1 μM SM ligand in the environment. The rates are normalized such that the peak of BLM-only rate is set to 1.0 and other rates are scaled accordingly. The distribution of rates is obtained by bootstrapping of rates obtained from single molecule traces. The BLM and ATP concentrations were 50 nM and 10 μM, respectively.

**Figure 2.**
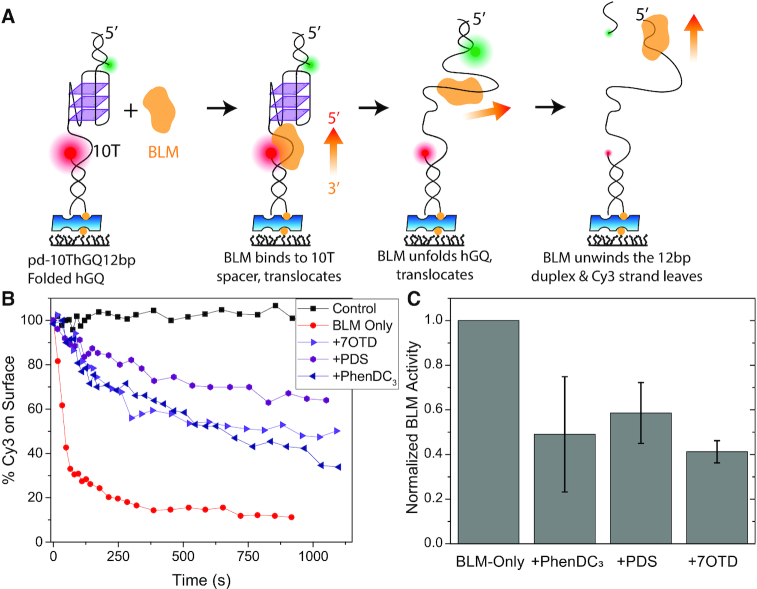
Quantification of cumulative BLM-mediated hGQ unfolding at 150 mM KCl and 2 mM MgCl_2_. (**A**) A schematic that summarizes the assay. BLM binds to the 10T spacer and proceeds to unfold the hGQ, followed by unwinding the 12-bp duplex. As a result, the Cy3-labeled strand leaves the surface while the Cy5-labeled strand remains bound via a biotin-neutravidin linker. The fraction of Cy3 molecules that leave the surface correlates with the number of hGQ molecules that have been unfolded by BLM at least once. (**B**) The fraction of Cy3 molecules that remains on the surface as a function of time. Control measurements (black squares) were performed in the absence of ATP, and represent the level of photobleaching in the assay, which is negligible. BLM-only (red circles) measurements do not contain any SM ligand and serve as reference to those with ligands. The concentration of SM is 1 μM for PhenDC_3_ (navy triangles), L1H1-7OTD (violet triangles), and PDS (purple hexagons). The lines between the data points are visual guides. BLM and ATP concentrations were 300 nM and 6 mM, respectively. (**C**) Fraction of Cy3 molecules that is removed from the surface 600 s after introducing the relevant agents. The data are presented in such a way that BLM-only activity is set to 1.0 (reference) and the activities in the presence of SM ligands are normalized accordingly. The results show that the BLM activity is suppressed by approximately 2-fold in the presence of SM ligands.

All DNA oligonucleotides (sequences are given in Supplementary Materials Table S1) were purchased from Integrated DNA Technologies (Iowa, USA) as HPLC or PAGE purified. The partial duplex DNA (pdDNA) constructs were formed by annealing individual strands in 10 mM Tris (for the data in Figure 1) or 150 mM KCl + 10 mM Tris (for the data in Figure 2) for 3 min at 90°C and slowly cooling down to room temperature over 2–3 h. The pH was 7.5 for both assays. The SM ligands used in this study are specific to GQ and are known to have negligible interaction with dsDNA (15,16,49).

The schematic of the DNA construct used for quantifying the impact of SM ligands on BLM dynamics, ss-hGQ10T, is shown in Figure 1A. The 5′-end is labeled with biotin and 3′-end with Cy5 fluorophore, which should have minimal impact of BLM loading (43). hGQ is expected to be formed in the middle portion of the construct with 10 thymidine (10T) overhang on the 3′-side, for BLM loading. To the 5′-side of hGQ lies a 4T spacer that is internally labeled with Cy3 fluorophore. This 4T spacer is shorter than the footprint of BLM (∼7 nt) (50). We did not observe any evidence of BLM loading and hGQ unfolding with such an overhang in our earlier work (51). Even if BLM loads transiently to this overhang, it would be expected to translocate in the 3′-to-5′ direction, away from the hGQ. Such events should not give rise to any hGQ unfolding and should not influence our analysis. A different DNA design was used for assessing the impact of SM ligands on cumulative BLM activity. This construct, pd-10ThGQ12bp (Figure 2A), contains two double-stranded DNA segments. One of these is 18 base pairs (bp) long and is attached to the surface while the other is 12-bp long and is on the opposite end. The hGQ is located between these two dsDNA segments.

### Experimental setup and single-molecule FRET (smFRET) assay

A prism-type total internal reflection fluorescence (TIRF) setup, equipped with an Olympus IX-71 microscope and an Olympus 60×, 1.20 NA water objective, was utilized to perform smFRET measurements. Single molecule sensitivity was achieved by an Andor Ixon EMCCD camera (iXon DV 887-BI EMCCD, Andor Technology, CT, USA). A λ = 532 nm green laser (SpectraPhysics Excelsior) was used to excite the donor (Cy3) fluorophores. Emissions from donor and acceptor (Cy5) fluorophores were separated by a dichroic mirror and projected on two halves of a CCD screen.

The quartz slides and glass coverslips were extensively cleaned, including Piranha etching, and coated with polyethylene glycol (PEG). 1% of PEG molecules were tagged with biotin to provide attachment points for biotinylated DNA molecules, which bind to biotin-PEG via neutravidin. In addition, the surface was coated with 1 mg/ml casein (overnight incubation) or 1% (v/v) Tween-20 (15-minute incubation before adding neutravidin) to reduce non-specific binding of BLM to the surface. To provide adequate statistics for the analysis, 250–350 DNA molecules per imaging area (∼5 × 10^3^ μm^2^) was targeted as the ideal density in most measurements.

To improve the photostability of Cy3 and Cy5, an oxygen scavenging system (0.1 mg/ml glucose oxidase, 0.02 mg/ml catalase, 0.8 mg/ml glucose) was added to the imaging buffer that contains Tris–HCl (50mM, pH 7.5), 2 mM Trolox, 0.1 mg/ml bovine serum albumin (BSA), 150 mM KCl and 2 mM MgCl_2_. Depending on the experiment, the imaging buffer also contained SM ligands, BLM, and ATP at desired concentrations. 1 mM dithiothreitol (DTT) was included in imaging buffer for measurements that included BLM. Before introducing BLM into a sample chamber, proper GQ folding was checked via smFRET histogram. We immediately started recording either short movies (15 frames) or long movies (1500–3000 frames) after introducing BLM into the sample chamber. The laser power and frame integration time (50 ms in Figure 1 and 200 ms in Figure 2) were adjusted depending on the goal of the measurement. All smFRET measurements were performed at room temperature, 23°C.

### Quantifying the impact of SM ligands on BLM activity

BLM activity was quantified in the presence or absence of SM ligands via two different approaches. In the first assay, we measured the rate of BLM-mediated hGQ unfolding (number of unfolding events per sec) with and without SM ligands. In the second assay, we measured the cumulative BLM activity where BLM unfolds hGQ at least once during our observation time. Both assays were performed under physiologically relevant salt conditions (150 mM KCl, 2 mM MgCl_2_ and pH 7.5), at which hGQ remains folded in the absence of BLM. To minimize variations in protein activity and surface quality, the measurements with SM ligands were performed immediately after those without SM ligands (will be referred to as ‘BLM-only’ case) in the same sample chamber (in the first assay) or the same slide (in the second assay).

In the first assay (Figure 1), hGQ unfolding by BLM is observed as a dip in the FRET efficiency below a certain threshold. The frequency of such dips was measured in the presence or absence of SM ligands. After completing the BLM-only measurements, the GQ molecules were reset with LiOH (52), and refolded by adding 150 mM KCl and 2 mM MgCl_2_. After a 15-minute incubation period under these conditions, the folding state is confirmed by smFRET measurements before BLM and SM ligands are introduced to the chamber. The hGQ is then incubated with 1 μM SM ligand for 15 minutes to ensure almost all hGQ are bound by a ligand. This is followed by adding 50 nM BLM, 10 μM ATP and 1 μM ligand, and start of data acquisition. As complex dynamic interactions are analyzed in this assay, the ATP and BLM were utilized at low enough concentrations to enable reliable detection of the unfolding events. This resulted in majority of smFRET traces not showing any transitions within our imaging time; however, facilitated analysis of those that did show transitions. Example of such traces that did not show transitions are shown in Supplementary Figure S2. For reliable detection of hGQ unfolding, folded states with high FRET levels are preferred, as these would result in a larger dip in E_FRET_ upon unfolding. Since parallel conformation (PC) is typically characterized by a higher FRET peak, we sought to increase the relative fraction of molecules that are in PC before BLM is added. As demonstrated in earlier studies, folding hGQ at room temperature, rather than at elevated annealing temperatures, increases the probability of kinetically trapping hGQ at the parallel conformation rather than the thermodynamically favored anti-parallel conformation (APC) (52,53). Therefore, hGQ was annealed in the absence of KCl just before the measurement. It was then immobilized on the chamber surface and folded at room temperature by adding a buffer that contains 150 mM KCl.

For the second assay (Figure 2), a DNA construct where a short duplex (12-bp) labeled with a donor Cy3 fluorophore is placed upstream of hGQ was designed. The hGQ has to be unfolded before BLM can reach the short duplex and unwind it. Unfolding of this 12-bp duplex releases the Cy3-labeled strand, which then diffuses away from the TIRF imaging volume. This assay was performed in the absence and presence of SM ligands. If a ligand imparts additional stability to hGQ, we expect to have more Cy3 molecules on the surface at any particular time. The fraction of Cy3 molecules that leaves the surface correlates with the fraction of hGQ molecules that have been unfolded by BLM at least once. In a physiological setting, this might be adequate for destabilization of GQ and enable Watson-Crick pairing between the G-rich and the complementary C-rich strands. Low intensity laser excitation and short exposures (15 frames at 200 ms integration time every several minutes) were used in time-lapse mode to minimize photobleaching. This is demonstrated by the approximately constant number of Cy3 molecules throughout the imaging period when ATP, which is required for duplex unwinding, is excluded from the imaging buffer (Figure 2B). Supplementary Figure S3 shows images demonstrating removal of Cy3 molecules from the surface while Cy5 molecules remain bound throughout the measurement. The smFRET measurements were performed at 150 mM KCl, 2 mM MgCl_2_ and pH 7.5, under which hGQ remains folded in the absence of BLM. BLM and ATP concentrations were 300 nM and 6 mM for this assay, respectively.

## RESULTS

We examined the impact of several prominent SM ligands, with different structural features, on hGQ-BLM interactions. An OTD similar to the one used in this study was estimated to remain bound to hGQ for ∼100 s (54). The dissociation constants of all of the SM ligands used in this study are in submicromolar to nanomolar range (18,55,56). To ensure that almost all hGQ molecules are bound by an SM ligand before interacting with BLM, we kept ligand concentration at 1 μM. Higher concentrations increase non-specific quenching induced by PDS and PhenDC_3_ (57,58), while significantly lower concentrations may result in negligible hGQ stabilization against helicase-mediated unfolding (58). As Δ*T*_m_ values for PDS, PhenDC_3_ and OTD’s have been characterized under different ionic conditions and different SM to hGQ concentration ratios, we performed CD thermal melting studies on hGQ and these SM ligands under consistent conditions. The data for different ligands are given in Supplementary Figure S1, and demonstrate significantly different levels of stabilization.

### Effect of SM ligands on GQ-BLM interaction dynamics

Figure 1A illustrates different steps of BLM-hGQ interactions. SM ligands were omitted from these schematics for clarity purposes. BLM binds to the 10T loading site and translocates in the 3′-to-5′ direction until it interacts with hGQ. If it unfolds hGQ, a sharp drop is observed in the FRET efficiency (E_FRET_). After BLM dissociates from DNA, hGQ refolds and the process restarts. Therefore, multiple unfolding events can take place for a particular DNA construct within our observation time.

In order to identify the unfolding events, a similar method to that we used in our earlier work (43,59) was employed. Briefly, we initially identified the FRET levels corresponding to the folded and unfolded states. The folded state is identified from the smFRET histogram before adding BLM (Figure 1B). This histogram shows three peaks at *E*_FRET_ = 0.12±0.02, 0.59±0.05 and 0.70±0.03. The peak at *E*_FRET_ = 0.12 is due to donor leakage into the acceptor channel and will be referred to as the donor-only (DO) peak. The standard procedure when reporting such histograms is to adjust the FRET axis such that the DO peak shifts to *E*_FRET_ = 0.0. However, as the analysis of dynamic unfolding events is performed on individual smFRET traces, which are not processed with this correction, the *E*_FRET_ levels corresponding to folded and unfolded states are determined without the DO correction. *E*_FRET_ = 0.59 represents the anti-parallel (or hybrid) folded conformation while *E*_FRET_ = 0.70 represents the parallel conformation (52). To identify the FRET peak that represents the unfolded state, we used a pdDNA construct with an overhang of 35 thymidines, pd-35T, which has similar length as ss-hGQ10T but does not form a secondary structure. FRET histogram for this construct is given in Supplementary Figure S2 (59). Based on these histograms, we established *E*_FRET_ ≤ 0.45 as a requirement for hGQ unfolding and *E*_FRET_ ≥ 0.55 for hGQ refolding. Figure 1C shows two example smFRET traces that illustrate the unfolding events starting from different folded FRET levels. The thresholds are marked with dashed red lines and successful unfolding events with green boxes. Supplementary Figure S4 shows several other example traces to illustrate the range of activity observed in these measurements.

We determined the average unfolding rate (s^−1^) by dividing the total number of observed unfolding events in hundreds of smFRET traces with the total observation time. The total time is the sum of individual observation times for each molecule, as obtained from smFRET traces (from the start of recording until photobleaching or end of recording whichever comes first). To illustrate, three transitions are observed in the top trace in Figure 1C within 54.3 s of observation time (until acceptor photobleaching). The unfolding rate for this molecule is 3/(54.3 s) = 0.055 s^−1^. Similarly, three transitions are observed in the bottom trace in Figure 1C within 29.5 s, resulting in a rate of 0.102 s^−1^. Table 1 reports the number of molecules, the number of unfolding transitions, the total observation time and the resulting average unfolding rate for each condition. The traces lacking any unfolding event were also included in the statistics and contributed to the total measured time. The error bars reported in Table 1 and the distribution in Figure 1D were obtained by bootstrap analysis. 20 000 bootstrapping sets were generated with a 95% confidence level (2.5–97.5%) from the single molecule data, and the average rate for each of these sets was calculated. To illustrate, let us consider the case of BLM-only reference for L1H1-7OTD where we measured the rates for *N* = 544 DNA molecules independently. To form one of the bootstrapping sets, 544 rates are selected randomly with replacement from among these rates, e.g. the rate corresponding to a particular DNA molecule might be selected more than once. The average rate for this set is then found. This process is repeated 20 000 times, resulting in 20 000 different averages for this particular condition. Figure 1D shows a histogram of these different averages. If the rates for individual molecules are similar to each other, a narrow distribution is obtained. If the individual rates demonstrate large variations, the distribution becomes wider. The standard deviation of a Gaussian fit to the bootstrapping distribution is reported as the uncertainty in the unfolding rate. Figure 1D shows the distribution of these average rates with the peak of the BLM-only condition normalized to 1.0. The BLM-only case for L1H1-7OTD is shown in Figure 1D to illustrate the distribution of the BLM-only rates. Supplementary Figure S5 shows the results of the bootstrapping analysis for each condition before normalization, as reported in Table 1. The unfolding rates reduced to 37%, 46% and 51% of their BLM-only case values in the presence of L1H1-7OTD, PDS and PhenDC_3_, respectively.

**Table 1.** Average BLM-mediated hGQ unfolding rates before and after adding SM ligands. The number of molecules describes the number of GQ molecules included in the analysis, and the total time is the cumulative observation time of all molecules. Number of events refers to the number of unfolding events we observed during the observation time. The uncertainties of the unfolding rates are based on the distribution of average rates obtained from bootstrapping analysis

Condition	# of Molecules	# of Events	Time (s)	Rate (1/s)
**BLM-Only Ref**	544	635	12241	0.052±0.008
**L1H1**-**7OTD**	590	256	13434	0.019±0.004
**BLM-Only Ref**	484	547	10074	0.055±0.006
**PhenDC_3_**	463	184	6493	0.028±0.003
**BLM**-**Only Ref**	560	429	7655	0.056±0.006
**PDS**	554	265	10066	0.026±0.004

### Effect of SM ligands on cumulative BLM-mediated hGQ unfolding activity

In this assay, unfolding of hGQ is imposed as a prerequisite for unwinding of a short duplex. Unwinding of the duplex releases a DNA strand that is labeled with Cy3, reducing the number of Cy3 molecules that remain on the surface. The more Cy3 strands are removed from the surface, the higher the BLM activity. In order to establish a reference level of activity, 300 nM BLM and 6 mM ATP were added to the sample chamber, without SM, and the number of Cy3 molecules on the surface was measured as a function of time. Similar measurements were then repeated in the presence of 1 μM L1H1-7OTD, PhenDC_3_ or PDS in different chambers. The ligands were added to the chamber 15 min before BLM to allow binding to hGQ. They were also added when with BLM and ATP are introduced to maintain a high ligand concentration throughout the measurement. Figure 2B shows the number of Cy3 (donor) molecules in the imaging area, normalized to a percent scale assuming an initial level of 100%, as a function of time for different SM ligands. While the data in the absence of SM ligands show the largest and steepest drop, the three SM ligands stabilize hGQ and result in higher fractions of surface-bound Cy3 molecules. To establish an estimate for the stabilization induced by ligands, we compared the number of Cy3 molecules that remain on the surface after 600 s (Figure 2C). Defining BLM activity in the absence of SM to be 1.0, this analysis showed that BLM maintains a relative activity of 0.41±0.05, 0.59±0.14, and 0.49±0.26 in the presence of L1H1-7OTD, PDS and PhenDC_3_, respectively. The error bars are standard deviations based on three independent measurements for each case. These results indicate that all SM ligands stabilize hGQ ∼2-fold against BLM.

## DISCUSSION

Even though the three SM ligands result in significantly different Δ*T*_m_ for hGQ, their impact on BLM activity showed only minor variations, which were within the uncertainties of the measurements. This raises interesting questions regarding whether Δ*T*_m_ values can be taken as reference for the impact of ligands on GQ stability against destabilizing proteins. In a cellular setting, interactions with proteins and competition with Watson-Crick pairing are the primary mechanisms that destabilize GQs. Even though it is reasonable to expect that Δ*T*_m_ induced by SM ligands would be a good estimator for GQ stability against Watson–Crick pairing, it is not clear whether this alone would be adequate to explain their impact against protein-mediated destabilization. Proteins may disrupt the stacking interactions between the SM ligand and GQ (58) before they unfold the GQ. In this case, the affinity of SM ligands to the GQ might be a more relevant parameter compared to ΔT_m_ they induce. However, it is also possible that a protein will interact with the GQ and the SM simultaneously and distort the GQ structure in the process before unfolding it. Such distortions in the GQ structure would likely depend on its thermal stability and influence the affinity of the SM to GQ as well. In this scenario, Δ*T*_m_ and affinity of SM to GQ are both significant and interdependent. The mechanistic details of these interactions likely depend on the structures of proteins and the SM ligands involved. Such details might be revealed with single molecule studies probing the underlying kinetics of these interactions. However, our data imply that factors beyond Δ*T*_m_ are also significant in determining the efficacy of SM ligands in stabilizing hGQ against BLM.

Our results are overall in quantitative agreement with earlier studies performed on different proteins and SM ligands using primarily ensemble measurements. Using a fluorescence quenching assay, Mendoza *et al.* reported that PDS and PhenDC_3_ (at 1 μM concentration) reduce Pif1-mediated hGQ unfolding activity to ∼38% and ∼63% of the activity in their absence, respectively (31). Li *et al.* quantified the impact of five different acridine derivatives on GQ unfolding activity of BLM and WRN using electrophoretic mobility shift assay (EMSA) (29). In this study, 6–7 μM SM ligand concentration was required to reduce BLM activity to 50% of its original value (IC_50_) in the case of less potent ligands, while 0.25–0.75 μM ligand concentration was adequate for the more potent ligands. Our results are similar to those on the more potent acridine derivatives used in that study. It should be mentioned that the inhibition observed by Li *et al.* was not specific to GQ as a similar level of inhibition was also observed for dsDNA. Wu and Maizels studied inhibition of *E. coli* RecQ helicase by porphyrin derivative NMM and reported an inhibition constant (the concentration of NMM at which RecQ-mediated GQ unfolding activity was inhibited by 50%) of 1.7 μM (30). Taking into account the 1 μM SM ligand concentration we used, 2–3-fold reduction in BLM activity is similar to the inhibition reported for RecQ.

Obtaining consistent rates of unfolding in different experiments was a significant challenge. These variations are influenced by various factors including (i) the complexity of data analysis for dynamic interactions; (ii) strong dependence of unfolding rates on surface quality; (iii) variations in protein activity, for which single molecule methods are highly sensitive to; and (iv) potential variations in the folding conformations of hGQ that result in different stabilities against BLM. Given these sources of variability, we focused on comparing the relative rates observed in the presence and absence of SM ligands as measured within the same DNA chamber, with the DNA molecules from the same annealing reaction, and the same batch of proteins. We will next discuss these issues in more detail to illustrate the range of challenges associated with these measurements and the insights we acquired from our efforts.

Several factors contribute to the complexity in data analysis. As we demonstrated in an earlier study, not every attempt by BLM results in complete unfolding of hGQ (43). Nevertheless, these attempts resulted in significant reduction in FRET. Our remedy for whether a reduction in FRET should be counted as an unfolding event was to establish optimal threshold FRET levels that represent the unfolded and refolded states, as indicated in Figure 1. However, these threshold values cannot be perfect as there is overlap in the FRET distributions of the folded and unfolded states. In addition, we occasionally observed very fast transition events with only a single data point below the threshold *E*_FRET_, which could be an unfolding event or just a signal fluctuation. For such cases, we adopted a conservative approach and did not consider such cases as unfolding events. Even though these criteria resulted in more consistent analysis results, we still observed about 25% variation in the obtained rates when the same data set was analyzed by different researchers independently. The consistency was not better with bias-free computational methods, such as Hidden-Markov model-based algorithms, e.g. ebFRET (60), unless a large majority of the traces were excluded from the analysis. The gradual nature of transitions to the unfolded state, instead of the sharp transitions typically sought by these algorithms, the need for establishing thresholds for considering a change in FRET as a complete unfolding event, and the challenge of distinguishing very short duration transitions from the typical noise in the data were some of the challenges while using these programs.

Surface quality and protein activity were other variables in these measurements. In addition to Piranha cleaning and PEG passivation that are considered to result in high-quality surfaces, we also incorporated additional casein (1 mg/ml) or Tween-20 (1% v/v) coatings as these steps resulted in higher and more consistent BLM activity. However, we still observed significant variations in the unfolding rates among different channels and slides prepared in different days. The propensity of BLM and ligand adsorption to the surface is probably a major factor in this variability. Single molecule methods are in general highly sensitive to variations in protein activity, especially at the relatively low concentrations used in our measurements. Even though a consistent protocol was followed for how proteins are stored and thawed before measurements, there might still be variations in their activity. SM ligand adsorption to the surface has been reported to result in fluorescence quenching due to interactions of the fluorophores with the ligands that are adsorbed to the surface (58). These non-specific effects could be reduced by using lower ligand concentrations (∼100 nM instead of 1 μM). However, at these lower concentrations, helicases were reported to easily displace the SM ligands, making them ineffective at stabilizing the GQ (58).

Another potentially significant issue is the folding conformations, as hGQ is known to attain different conformations depending on annealing and storage conditions. These different conformations are known to have different characteristics in terms of stability against a protein and folding kinetics (53,61,62). We investigated whether SM ligands give rise to conformational changes under our assay conditions using circular dichroism (CD) and smFRET. For CD measurements, the samples were prepared following the same annealing and sample preparation protocol as in smFRET assay and all measurements were performed under 150 mM KCl and 2 mM MgCl_2_. In the absence of SM ligands, hGQ shows a mix of PC and APC characterized by a peak at 290 nm and a hump at 260 nm (Supplementary Figure S6). This is consistent with the two folding peaks, corresponding to PC and APC, observed in smFRET histogram (Figure 1B). Upon adding the SM ligands to already folded hGQ molecules and incubating for 15 minutes, we observe a shift towards APC for all three ligands, characterized by a peak at 290 nm and a trough at 260 nm (Supplementary Figure S6). In the absence of SM ligands, hGQ makes a similar transition from PC to APC over several hours (52,53). Therefore, SM ligands have significantly accelerated this transition to the thermodynamically more stable conformation. On the other hand, if hGQ is already folded in APC, the SM ligands do not induce a further change in conformation as demonstrated by smFRET measurements (Supplementary Figure S7). The hGQ molecules were annealed in KCl for these measurements, which resulted in predominantly APC. Adding SM ligands to these samples did not result in a significant change in conformations, except a broadening of peaks in PDS. Therefore, we conclude that SM ligands accelerate the transition from PC to APC but they do not result in a significant change if hGQ molecules are already in APC.

The second assay presented on this topic (Figure 2), was developed as an independent way of quantifying the impact of SM ligands on BLM activity where some of the complications associated with analyzing dynamic traces could be avoided. Similar precautions were taken to minimize the variability due to the surface, proteins, or folding patterns and the observed rates were normalized with respect to the BLM-only activity, which was measured on the same slide. The primary advantage of this assay is the simplicity of the data analysis. Rather than analyzing single molecule traces with complicated dynamics, only the number of Cy3 molecules that remain on the surface were identified and counted by a computer program. This is an indirect measurement of BLM activity, and is based on the assumption that a Cy3 molecule that remains on the surface is a reporter for an hGQ that has not been unfolded by BLM. Clearly, this assumption will not always be satisfied, as there would be cases where BLM will dissociate from the DNA after it unfolds hGQ and before it completely unwinds the 12-bp duplex. Nevertheless, the correlation that formed the basis of this analysis should still hold and hGQ stabilized by SM ligands should result in a higher fraction of surface-bound Cy3 molecules. Despite the assumptions, complications, and imperfections of both assays, it is encouraging to observe that the results are within reasonable quantitative agreement with each other, e.g. these prominent SM ligands decrease BLM-mediated hGQ unfolding by 2–3-fold.

## CONCLUSION

The impact of several prominent small molecule ligands (PDS, PhenDC_3_ and L1H1-7OTD) on BLM-mediated hGQ unfolding activity was investigated using two independent single-molecule approaches. The SM ligands resulted in 2–3-fold decrease in BLM activity when the rate of dynamic unfolding events was used as the metric. Independently, we estimated the ligand-induced stabilization against BLM to be ∼2-fold when hGQ unfolding was implemented as a pre-requisite for the unwinding of a short duplex. These results provide complementary perspectives to quantifying SM ligand efficacy to commonly used thermal melting studies. A more complete understanding of the mechanisms underlying these interactions would likely require insights from both of these approaches in addition to high-resolution structure studies. This study helps this cause by introducing two independent assays that could be adapted to study other such systems. Finally, this work also provides an estimate for the level of induced stability that could be expected in a physiological setting where GQs are unfolded or destabilized by proteins.

## Supplementary Material

gkz803_Supplemental_File
