# Supplementary material for: Quantifying the impact of small molecule ligands on G-quadruplex stability against Bloom helicase
Source: Nucleic Acids Res. 2019 Sep 23;47(20):10744–53. doi: 10.1093/nar/gkz803 (PMC6847008; doi:10.1093/nar/gkz803)
Supplement: gkz803_Supplemental_File [file gkz803_supplemental_file.pdf]

Supplementary Data

Quantifying the impact of small molecule ligands on G-quadruplex stability against Bloom helicase

Parastoo Maleki[1], Golam Mustafa[1], Prabesh Gyawali[1], Jagat B. Budhathoki[1], Yue Ma[2], Kazuo Nagasawa[2], Hamza Balci[1,*]

[1] Department of Physics, Kent State University, Kent, OH, 44242, USA

[2] Department of Biotechnology and Life Science, Tokyo University of Agriculture and Technology, Koganei, Tokyo 184-8588, Japan

*To whom correspondence should be addressed. Tel: +1 330 672 2577; Fax: +1 330 672 2959; Email: hbalci@kent.edu

Construct	Sequence (5′–3′)	Stem Complement
ss-hGQ10T	Biotin-TT-Cy3-TT GGGTTAGGGTTAGGGTTAGGG T_{10}-Cy5	
pd-35T	Cy3- T_{35}**TGG CGA CGG CAG CGA GGC**	Strand 1
pd-10ThGQ12bp	*GTA CGA TCG CAG* TTG GGT TAG GGT TAG GGT TAG GG T_{10} **TGG CGA CGG CAG CGA GGC**	Strand 1 & 2
hGQ_CD	TTGGGTTAGGGTTAGGGTTAGGGTT	
Strand 1	Biotin- **GCC TCG CTG CCG TCG CCA**-Cy5	
Strand 2	Cy3-*CTG CGA TCG TAC*	

Table S1: DNA sequences used for this study. The sequences that form the 18-bp duplex stem are shown in bold fonts. The sequences forming GQ are underlined. Italic sequences form the 12-bp duplex.

Circular Dichroism (CD) Thermal Melting Studies

In order to characterize the impact of the small molecule (SM) ligands on thermal melting of human telomeric GQ (hGQ) under consistent assay conditions, we performed CD thermal melting studies at 20 mM KCl, 2 mM $MgCl_2$, and pH 7.5. This lower KCl concentration (compared to 150 mM KCl used in smFRET measurements) was selected in order to ensure the transition from folded to unfolded hGQ would complete within our temperature range (T<95 °C) even in the presence of SM ligands. The construct "hGQ_CD" was used for these measurements (see Table S1 for sequence). SM ligand to hGQ ratio of 5:1 was used for all constructs. Due to specific circumstances associated with hGQ and its multiple conformations, we performed the CD thermal melting studies after hGQ reaches the thermodynamically more stable anti-parallel conformation. This is necessary to ensure that the change in the ellipticity is due to thermal melting rather than a conformation change from parallel to antiparallel. Therefore, the hGQ molecules were annealed in 20 mM KCl and incubated under these conditions overnight. The CD thermal melting measurements were performed after incubating the hGQ constructs with SM ligands for 1 hr at room temperature. The ellipticity at 292 nm was tracked as a function of temperature for all constructs as this was the peak value for the antiparallel conformation. As demonstrated in Figure S1, the three SM ligands result in significantly different shifts in thermal melting point (ΔT_m), which was determined based on a Gaussian fit to the first derivative of the data shown in Figure S1. The T_m values that result from this analysis are: T_m= 55.9 °C for hGQ in the absence of SM ligands; T_m= 61.6 °C in the presence of PDS; T_m= 63.6 °C in the presence of L1H1-7OTD; T_m= 72.0 °C in the presence of $PhenDC_3$. Based on these, ΔT_m= 5.7 °C for PDS; ΔT_m=7.7 °C for L1H1-7OTD; and ΔT_m=16.1 °C for $PhenDC_3$ under our assay conditions.

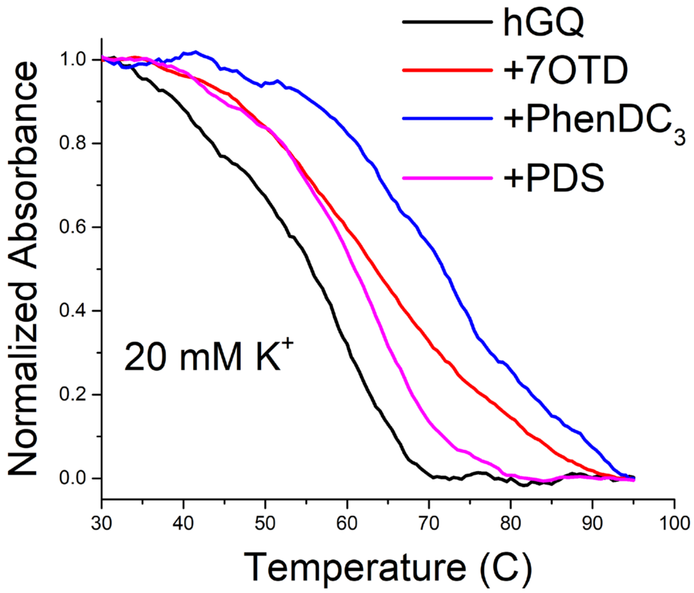

Figure S1: CD thermal melting studies on hGQ in absence of SM ligands or presence of PDS, L1H1-7OTD, and $PhenDC_3$. The ellipticity at 292 nm was tracked as a function of temperature. All measurements were performed at 20 mM KCl, 2 mM MgCl2 and pH 7.5. The ligand to hGQ ratio was fixed at 5:1 for all three ligands.

Reference smFRET Measurements and Example Traces

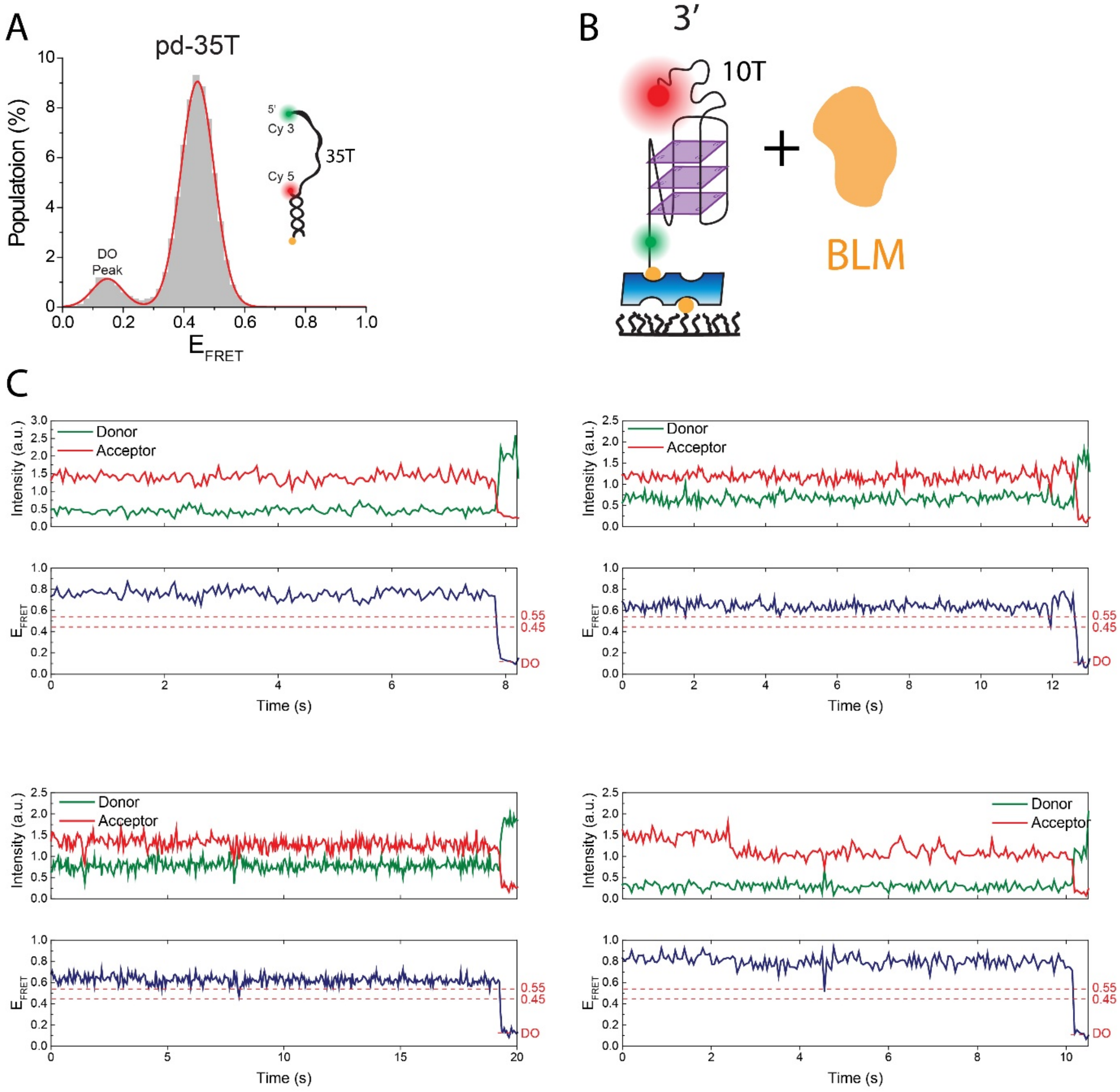

Figure S2. (A) smFRET histogram for pd-35T construct at 150 mM KCl and 2 mM $MgCl_2$, which is used as a reference for the unfolded hGQ state. The relevant peak for the analysis is at E_{FRET} = 0.45, which has been taken as a threshold value for the unfolded state of hGQ construct. The donor only peak (E_{FRET} = 0.12) has been kept in the histogram with the original scale as this is the level observed in the single molecule traces. (B) Schematic of the assay employed for traces shown in (C). (C) Example traces that do not show transitions in the presence of BLM and ATP. Such traces constitute the majority of traces and are included in the analysis as they contribute to the total observation time. The bottom left trace shows an event that goes below E_{FRET}=0.45 around time=8 s. However, this was not counted as an unfolding event since it was a single point below the threshold.

Images Demonstrating Removal of Cy3 from the Surface

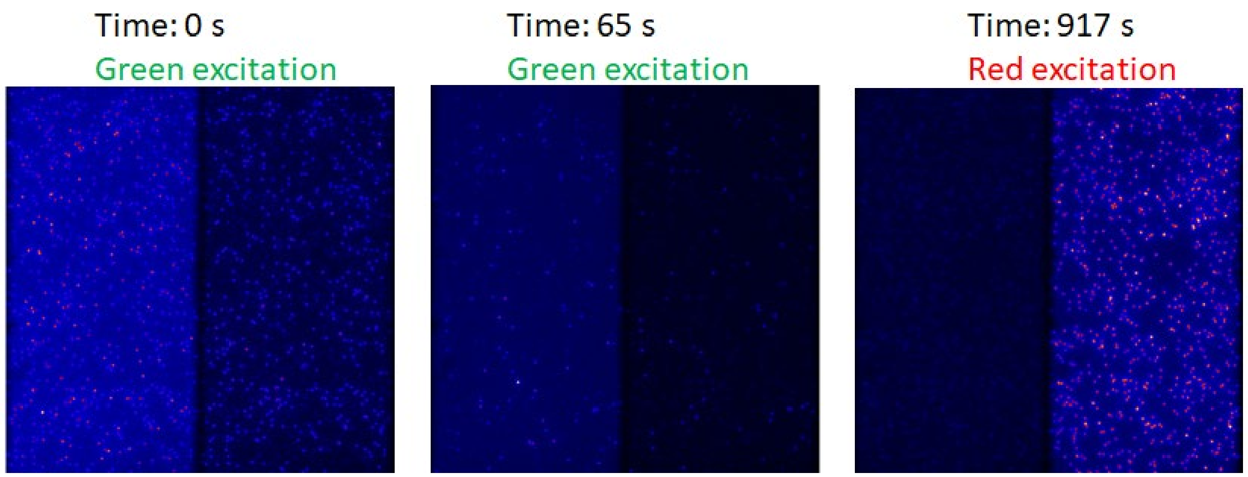

Figure S3. Images illustrate the reduction in the number of donor molecules (left half of the screen) as a function of time while the acceptor molecules (right half of the screen) remain on the surface. The leftmost image shows the surface with green excitation at the beginning of measurement where significant number of molecules are visible in both the donor and acceptor channels. After 65 s, the number of donor molecules already shows significant reduction. The acceptor molecules remain on the surface and have not photobleached even after 917 s, as shown with the image on the right taken when acceptor molecules were directly excited with a red laser.

smFRET Traces Demonstrating Unfolding Dynamics

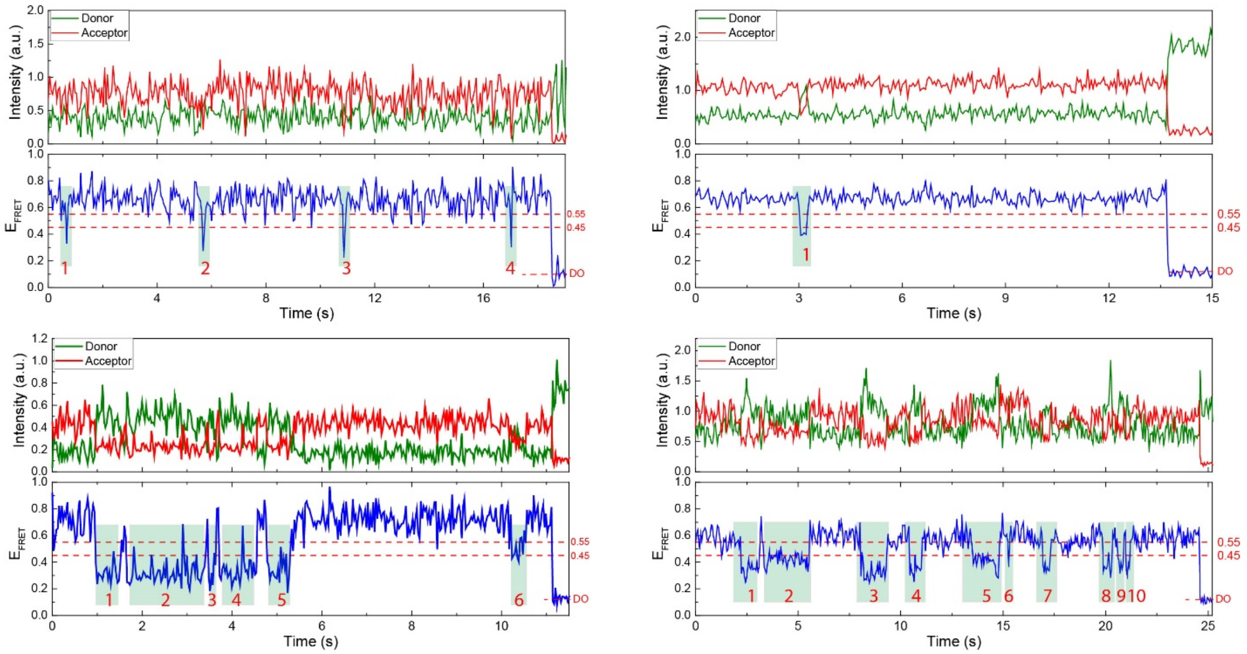

Figure S4. Example smFRET traces demonstrating the range of different features observed during the dynamic interactions of BLM, hGQ, and small molecules. The sections that represent hGQ unfolding events are marked with green boxes and numbered. The red dashed lines are the threshold values for hGQ unfolding ($E_{FRET} \leq 0.45$) and refolding ($E_{FRET} \geq 0.55$). The BLM and ATP concentrations were 50 nM and 10 μM, respectively. KCl concentration was 150 mM and $MgCl_2$ concentration was 2 mM.

Results of Bootstrapping Analysis before Normalization

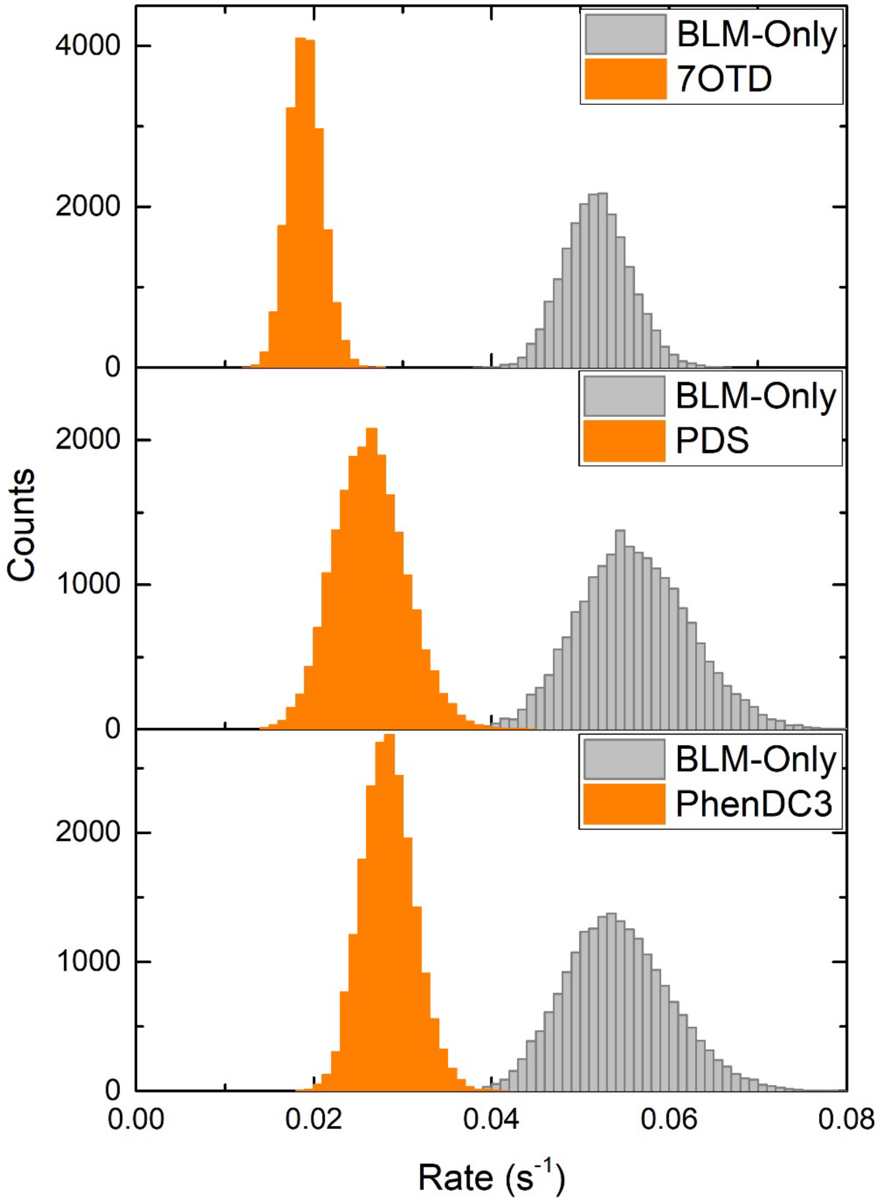

Figure S5. The distribution of mean BLM-mediated hGQ unfolding rates in the absence and presence of 7OTD (top), PDS (middle), and PhenDC$_3$ (bottom) as obtained from bootstrapping analysis. 20,000 bootstrapping sets with 95% confidence level (2.5%-97.5%) were generated for each of the peaks in the histograms. BLM and ATP concentrations were 50 nM and 10 μM, respectively. KCl concentration was 150 mM and $MgCl_2$ concentration was 2 mM.

CD Measurements Investigating hGQ Conformation Change Due to SM Ligands

In order to investigate whether SM ligands influence the folding conformation of hGQ, we performed measurements under identical assay conditions as those used in smFRET assay. The hGQ molecules were annealed in the absence of KCl and incubated in 150 mM KCl for 15 minutes. The SM ligands were added after this 15-minute incubation in KCl. The CD measurements were performed 15 minutes after the SM ligands are added. In the absence of SM ligands, hGQ shows a mix of parallel and anti-parallel conformations characterized by a peak at ~292 nm and a shoulder at ~260 nm. After SM ligands are added, the shoulder at 260 nm is eliminated and the peak at ~292 nm becomes more prominent. These are consistent with SM ligands inducing a parallel conformation under our assay conditions.

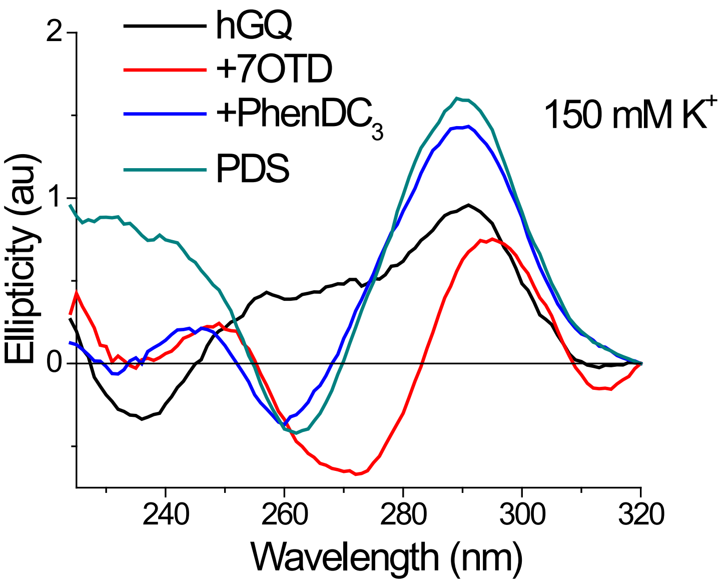

Figure S6: Circular dichroism measurements on hGQ molecules before and after adding SM ligands. The ligands primarily induce a transition to the anti-parallel conformation.

smFRET Measurements Investigating Conformation Change Due to SM Ligands

We performed smFRET measurements on hGQ molecules (same construct as that used in Figure 2) after they reach a predominantly antiparallel conformation in the absence of presence of SM ligands. The hGQ molecules were annealed in 150 mM KCl and incubated in this salt until the majority of molecules show antiparallel conformation, represented by the FRET peak in around $E_{FRET} \approx 0.48$. Upon addition of 1 μM SM ligands, and incubating for 15 minutes we did not observe a significant change in the distribution for any of the SM ligands, except for a broadening of the peaks for PDS.

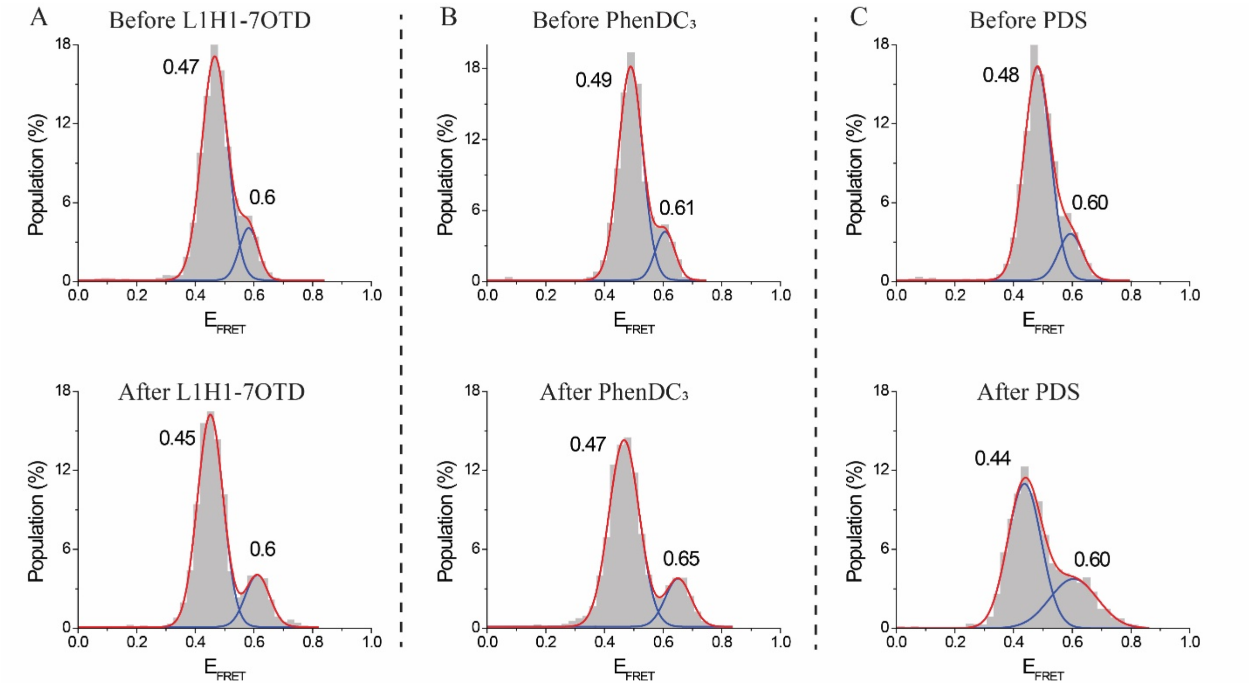

Figure S7: smFRET histograms before (upper panels) and after adding 1 μM SM ligand (lower panels). (A) L1H1-7OTD); (B) PhenDC$_3$; (C) PDS. All measurements were performed at 150 mM KCl and 2 mM $MgCl_2$. The numbers on the histograms indicate the peak positions. The histograms essentially remain unchanged upon adding the SM ligand, except in the case of PDS, which results in broadened peaks. These results suggest that if hGQ molecules are in predominantly antiparallel conformation (lower FRET peak), the folding is not significantly influenced by the SM ligands under our assay conditions.
